# A meta-analysis of closed reduction percutaneous pinning and open reduction with pin fixation of pediatric humeral lateral condylar fracture

**DOI:** 10.3389/fped.2023.1205755

**Published:** 2023-06-30

**Authors:** Chao Meng, Zhen Meng, Xin Huang, Fenghua Zhao, Qun Yang

**Affiliations:** ^1^ Department of Pediatric Surgery, The Affiliated Tengzhou Central People’s Hospital of Xuzhou Medical University, Tengzhou, China; ^2^ Department of Quality Control, Tengzhou Hospital of Traditional Chinese Medicine, Tengzhou, China; ^3^ Department of Infectious Diseases, The Affiliated Tengzhou Central People’s Hospital of Xuzhou Medical University, Tengzhou, China

**Keywords:** closed reduction, open reduction, humeral lateral condylar fracture, child, metaanalysis

## Abstract

**Objective:**

To compare the effectiveness and safety of closed reduction percutaneous pinning vs. open reduction with pin fixation to treat the pediatric humeral lateral condylar fracture.

**Methods:**

Studies comparing closed reduction percutaneous pinning vs. open reduction with pin fixation for treating pediatric lateral humeral condyle fractures were found by searching Pubmed, Embase, the Cochrane Library, and Web of Science databases, including randomized/non-randomized controlled, retrospective case-control, and prospective cohort studies. Furthermore, quality evaluation and data retrieval were conducted after the literature review. A meta-analysis was performed using RevMan 5.4 software to compare both groups' outcome measures.

**Results:**

This Meta-analysis incorporated eight studies with 856 cases. The Meta-analysis found no significant difference in functional outcomes, superficial infection, deep infection, poor fracture union, avascular necrosis of the humeral capitulum, or lateral spur formation between groups. However, the status of unaesthetic scars in the closed reduction percutaneous pinning group was superior.

**Conclusions:**

For pediatric humeral lateral condylar fracture surgical therapy, the efficacy and safety of closed reduction percutaneous pinning vs. open reduction with pin fixation were not significantly different; closed reduction percutaneous pinning offered the benefit of eliminating unaesthetic scar. However, further high-quality research is required to verify the conclusions of this Meta-analysis.

**Systematic Review Registration:**

https://www.crd.york.ac.uk/prospero/#myprospero, identifier CRD42023392451.

## Introduction

Humeral lateral condylar fractures (HLCF) are children's second most prevalent kind of elbow fracture, accounting for 17%–20% of all fractures (1–5). Since the fracture is intra-articular, anatomical repositioning must be performed afterward. However, anatomical repositioning is performed mainly by surgical intervention (with pin fixation or cannulated screw fixation) for significantly displaced or rotated HLCF. Open reduction with pin fixation (ORPF), a conventional surgical technique, is highly effective (6–8). Pediatric HLCF has recently been treated using closed reduction percutaneous pinning (CRPP). CRPP has particular advantages regarding functional outcomes, complications, and unaesthetic scars (9, 10).

Nevertheless, some researchers maintain that ORPF offers more substantial advantages, including better repositioning and fewer complications (11). As a result, perspectives on the surgical technique for pediatric HLCF are mixed. To provide clinical decision-making guidance, this systematic review and meta-analysis compared the functional outcomes, complications (superficial infection, deep infection, avascular necrosis of the humeral capitulum (ANHC), poor fracture union, and unaesthetic scars), and lateral spur formation (LSF) between the two surgical approaches.

## Methods

### Search strategy

(“humeral fracture, distal” [MeSH term] OR “humeral lateral condylar fracture” OR “humerus lateral condylar fracture” OR “lateral condylar fracture of the elbow”) AND (child[MeSH term] OR children OR pediatric) were searched for in the electronic databases Pubmed, Embase, Cochrane Library, and Web of Science without regard to ethnicity or language from inception to February 10, 2023. Moreover, references to all contained literature were evaluated.

### Selection criteria

Inclusion criteria: (1) All controlled studies of ORPF and CRPP for treating HLCF in children, including randomized controlled, nonrandomized case-control, retrospective case-control, and prospective cohort studies. (2) All patients with clinical manifestations and imaging diagnoses of HLCF were under 16 years old and had obvious surgical indications, regardless of race, country, or gender. (3) Outcomes included one or more of the following: functional outcomes, complications (superficial infection, deep infection, ANHC, poor fracture union, unaesthetic scar), and LSF.

Exclusion Criteria: (1) Case reports, case series, reviews, and correspondence. (2) The presence of other morbidities can have a significant effect on therapy and prognosis, multiple or open fractures. (3) HLCF is treated non-operatively or surgically without using a Kirschner wire. (4) The clinical outcomes were not reported in the studies.

### Study selection

A single reviewer applied the selection criteria to all publications obtained by the search strategy. Two reviewers evaluated the inclusion of identified studies. A third researcher could be consulted if disagreements concerning the literature's suitability arise. The titles of publications and authors' identities were not hidden throughout the selection procedure.

### Quality assessment

Using the Methodological Index for Trials (MINORS) with a total score of 12 as inclusion criteria, the methodology quality and bias risk of nonrandomized controlled studies were assessed by two researchers. The same two researchers utilized the Newcastle-Ottawa Scale (NOS) to assess prospective cohort and retrospective case-control studies, with a maximum score of 8, focusing on population selection, comparability, and outcome/exposure, and only studies with a score of 5 were included.

### Data extraction

Two researchers independently extracted the obtained data, and in the case of a dispute, the data were resolved by consultation, if required, with the participation of third researchers. Extracts included background information such as the first author's name, publication year, nation, sample size, type of research, and follow-up.

The significant outcome of interest was the functional outcome, graded with a rating of excellent, good, moderate, or poor. We primarily assessed the excellent and good rates of joint function following two surgical procedures. Secondary outcomes included superficial infection, deep infection, ANHC, poor fracture union, unaesthetic scar, and LSF. The number of individuals who dropped to follow-up was also a study characteristic. A solitary reviewer collected data on data abstraction forms from the selected studies.

### Statistical analysis

A meta-analysis was proposed if populations, interventions, and outcome assessments in trials were clinically homogenous. For statistical analysis, Review Manager 5.4 was employed. The odds ratio (OR) and 95% confidence interval (CI) were computed for a binary variable. Using the chi-square and *I^2^* tests, heterogeneity between selected literature was assessed. The pooled ORs were calculated using the fixed effects model if the associated *P* value was more than 0.05 or the *I^2^* was less than 50%, indicating decreased heterogeneity across studies; otherwise, the random-effects model was utilized. In cases of significant heterogeneity, sources of heterogeneity were investigated by either modifying the effect model used for the meta-analysis or removing each search individually.

## Results

### Search results

**Figure 1:**
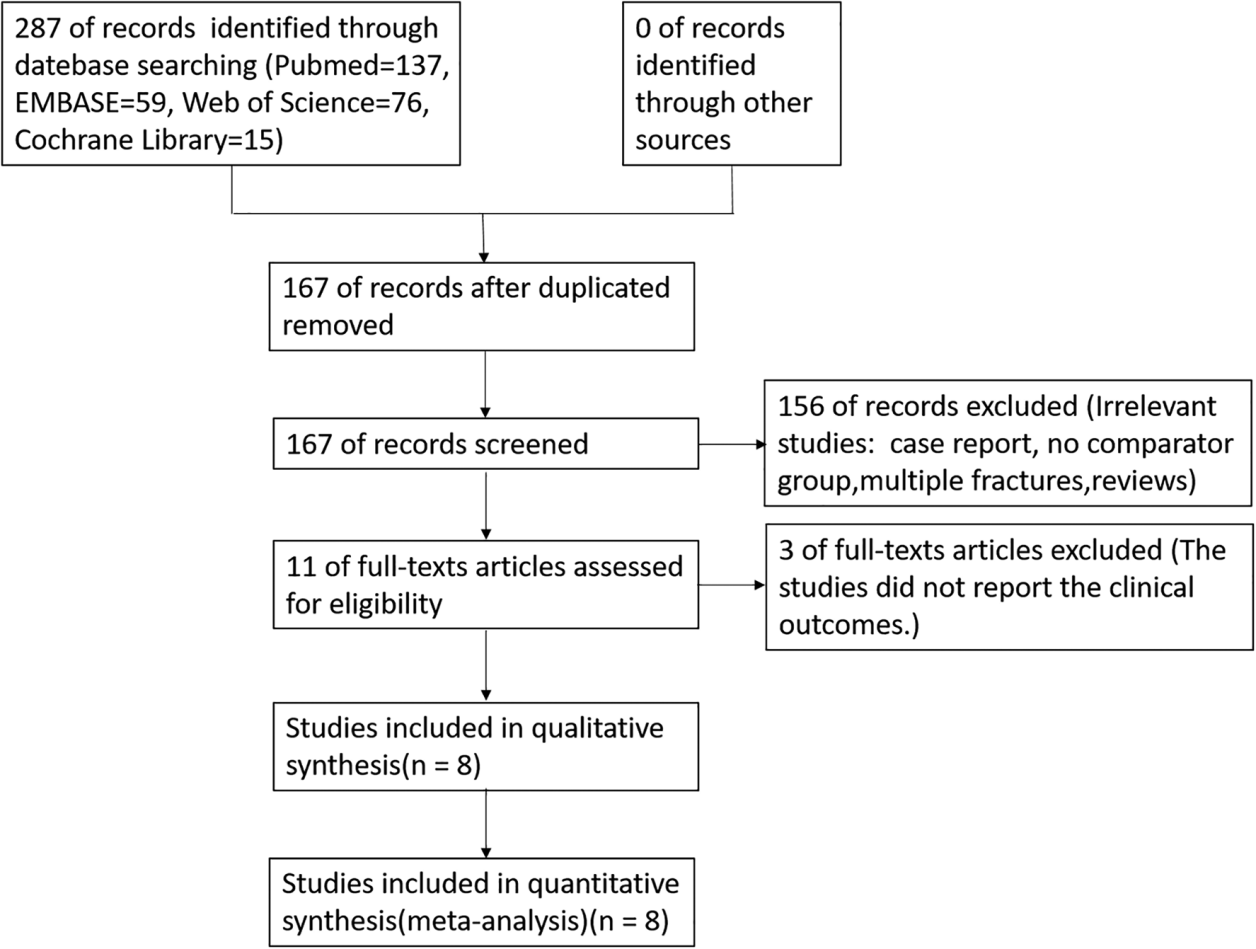
Flowchart of literature search and selection.

Initially, 287 articles were retrieved, including 137 through Pubmed, 59 through Embase, 76 through Web of Science, and 15 through Cochrane Library. 167 remained after filtering for duplicates using Endnote software. After reading the title pages and abstracts, 156 articles were removed following selection criteria, leaving 11 papers. After reviewing the entire manuscript, eight articles were preserved (12–19), as shown in Figure 1.

### Study characteristics

With a total of 856 children, there were six retrospective case-control studies, one prospective cohort study, and one nonrandomized controlled trial, with 617 cases in the ORPF group and 239 cases in the CRPP group. In the included publications, all cases were recovered for follow-up. Table 1 displays the clinical characteristics of the selected literature.

**Table 1:** Clinical characteristics of selected literature.

Study (years)	Mean age (years)		Gender		Simple size		Follow-up (months)	Classification (Criteria)
	CRPP	ORPF	Male	Female	CRPP	ORPF		
L Weng 2022 (12)	4.90 ± 2.33	5.39 ± 2.03	26	20	10	31	≥6	Song
C Justus 2017 (13)	5.16 ± 2.15	5.29 ± 2.27	113	59	31	141	4.54	Jakob
MI Kotb 2013 (14)	6.5 ± 1.75	6 ± 1.5	37	23	18	6	12	Song
M Silva 2015 (15)	5 ± 3.38		122	69	28	163	≥3	Milch
AT Pennock 2015 (16)	4.5 ± 2.00		45	29	23	51	6	Milch
Y Liu 2022 (17)	4.4 ± 2.08		53	22	39	36	≥12	Jakob
L Xie 2021 (18)	5.3 ± 2.5	5.1 ± 2.2	68	39	45	62	13.9	Song
KH Koh (19)	5.0 ± 2.61	5.1 ± 2.42	95	41	33	103	19.8	Milch/Jakob

CRPP, closed ruction percutaneous pinning; ORPF, open reduction with pin fixation.

Almost all of the selected literature implies that when CRPP fails, it should be converted to ORPF, but only three studies record examples of failure, for a total of 21 failed CRPPs, which we present in Table 2.

**Table 2:** Clinical outcomes of conversions to ORPF.

Study (years)	Converted to ORPP	Surgery duration (minutes)	Fracture healing (weeks)	Complications	Functional outcome (“excellent” or “good”)	Percent conversions to ORPP
L Weng 2022 (12)	5	81 ± 8.43	6.20 ± 0.84	1	4	33.33%
Y Liu 2022 (17)	3	NR	NR	NR	NR	7.14%
L Xie 2021 (18)	13	70.2 ± 8.9	NR	4	13	22.41%

NR, not report; ORPF, open reduction with pin fixation.

### Methodological assessment of study quality

The MINORS appraisal scores for the nonrandomized control trials included are shown in Table 3, and a single nonrandomized controlled trial received a scale score of 15. The NOS appraisal scores for retrospective case-control or prospective cohort studies are presented in Table 4, and one prospective cohort study had a score of 6. In contrast, six retrospective case-control studies received scores higher than 5.

**Table 3:** MINORS appraisal scores for the nonrandomized control trials.

Quality assessment for nonrandomized trials	MI Kotb 2013 (14)
A clearly stated aim	2
Consecutive patients included	2
Prospective data collection	0
Appropriate end points for the aim of the study	1
Unbiased assessment of the study end point	0
A follow-up period appropriate for the study aim	1
Less than 5% loss to follow-up	2
Prospective sample size calculation	0
An adequate control group	1
Contemporary groups	2
Baseline equivalence of groups	2
Adequate statistical analyses	2
Total score	15

**Table 4:** NOS appraisal scores for retrospective case-control or prospective cohort studies.

Study (year)	Design	Selection	Comparability	Exposure	Outcome	NOS Score
L Weng 2022 (12)	RCS	**	*	**		*****
C Justus 2017 (13)	RCS	**	*	***		******
M Silva 2015 (15)	PCS	***	*		**	******
AT Pennock 2015 (16)	RCS	***		***		******
Y Liu 2022 (17)	RCS	**	*	***		******
L Xie 2021 (18)	RCS	***	*	**		******
KH Koh (19)	RCS	**	*	***		*****

RCS, retrospective case-control studies; PCS, prospective cohort studies.

Each item's score is represented by a *, and one * equals one point.

### Results of the meta-analysis

#### Functional outcomes

**Figure 2:**
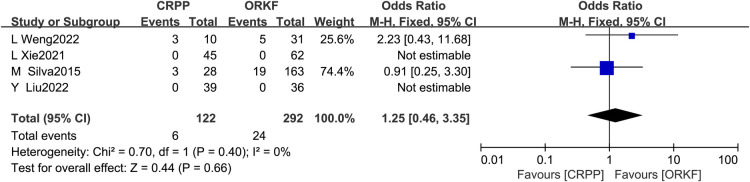
Forest plots of functional outcomes.

Four articles (12, 15, 17, 18) reported functional outcomes, with 122 cases in the CRPP arm and 292 cases in the ORPF arm. The test for heterogeneity revealed *I^2 ^*= 0%, *P *= 0.40, so a fixed-effects meta-analysis was performed, and the results [OR = 1.25, 95% CI (0.46, 3.35), *P *= 0.66] indicate that functional outcomes did not significantly differ between the two surgical procedures (Figure 2).

#### Superficial infection

**Figure 3:**
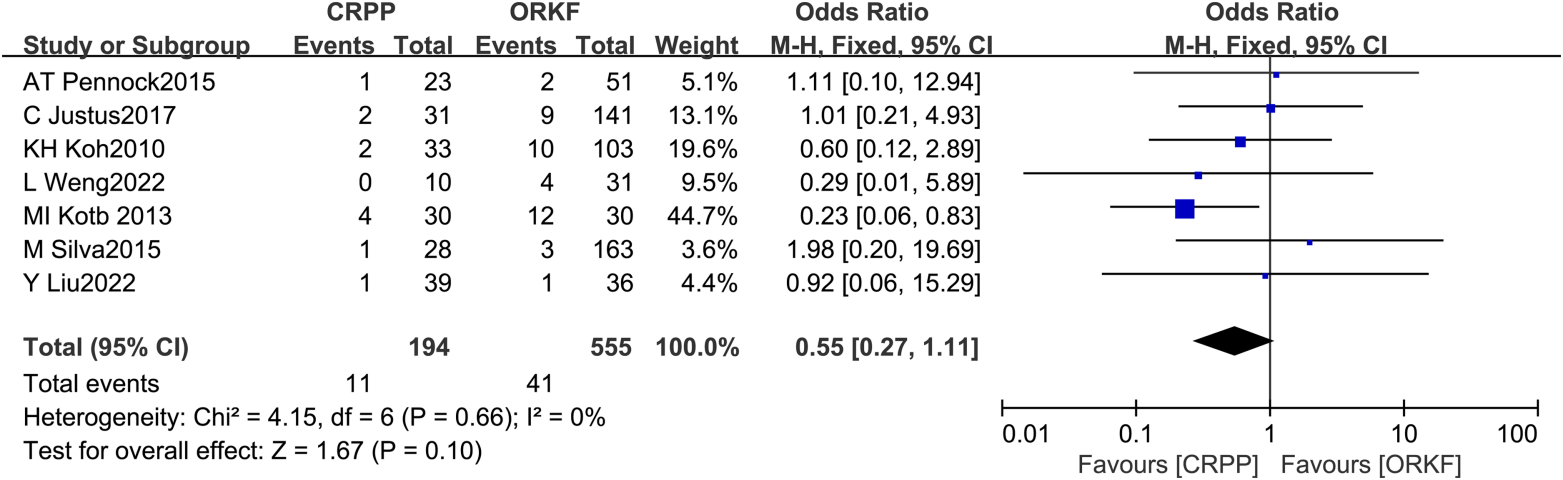
Forest plots of superficial infection.

Seven studies (12–17, 19) examined the prevalence of superficial infection in two groups, with 194 cases in the CRPP arm and 555 cases in the ORPF arm; the test for heterogeneity found *I^2 ^*= 0%, *P *= 0.66, necessitating a fixed effects model for Meta-analysis. In terms of superficial infection, we found no significant difference between both procedures [OR = 0.55, 95% CI (0.27, 1.11), *P *= 0.10] (Figure 3).

#### Deep infection

**Figure 4:**
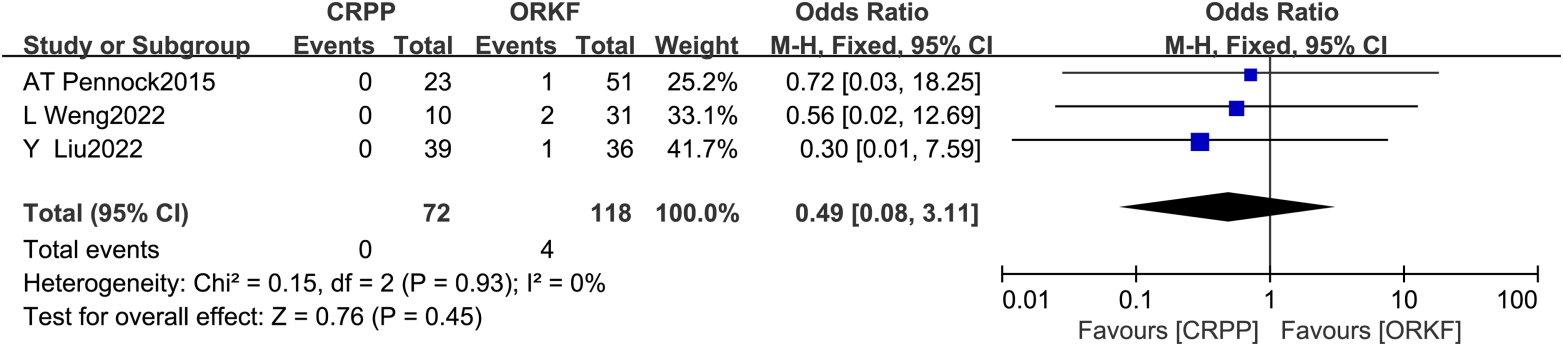
Forest plots of deep infection.

Three articles (12, 16, 17) recorded the occurrence of deep infection in two groups, with 72 cases in the CRPP arm and 118 cases in the ORPF arm; Fixed effects models were used for the meta-analysis because the test for heterogeneity revealed *I^2 ^*= 0% and *P *= 0.93. However, we found no significant difference between both procedures in deep infection. [OR = 0.49, 95% CI (0.08, 3.11), *P *= 0.45] (Figure 4).

#### Poor fracture union (delayed union, nonunion, and malunion)

**Figure 5:**
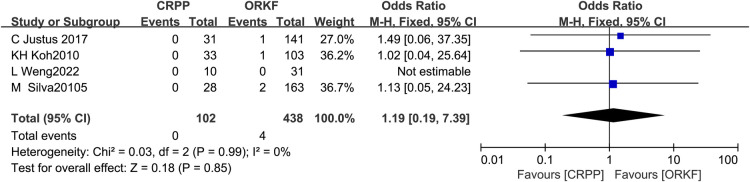
Forest plots of the poor fracture union.

Four articles (12, 13, 15, 19) reported poor fracture union in two groups, with 102 cases in the CRPP group and 438 cases in the ORPF group. The test for heterogeneity showed *I^2 ^*= 0%, *P *= 0.99, so a meta-analysis was performed employing a fixed effects model, and no significant difference was identified in poor fracture union between both procedures [OR = 1.19, 95% CI (0.19, 7.39), *P *= 0.85] (Figure 5).

#### ANHC

**Figure 6:**
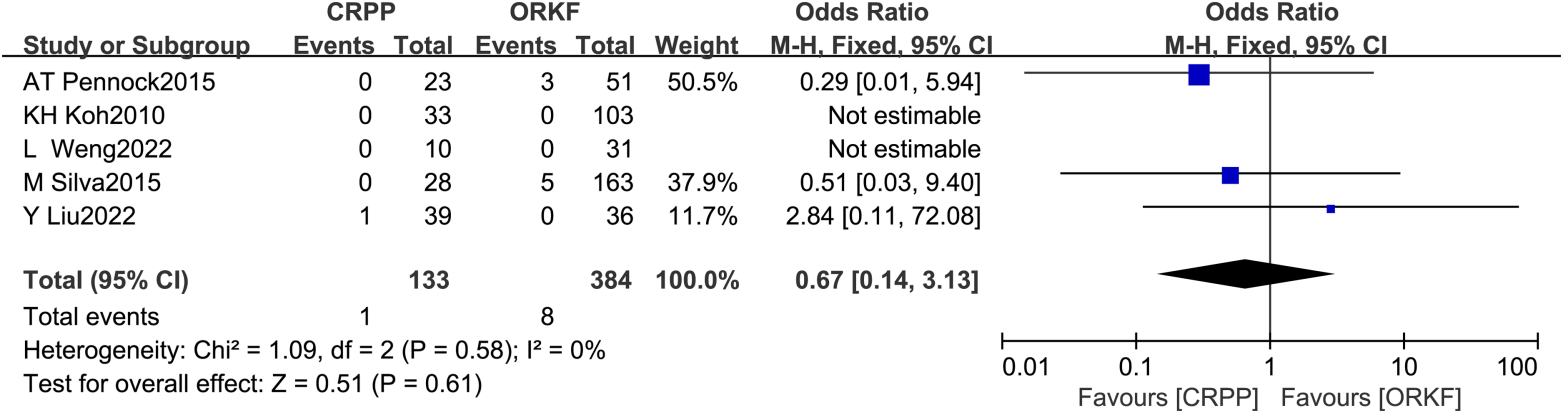
Forest plots of ANHC.

Five articles (12, 15–17, 19) reported ANHC, with 133 cases in the CRPP arm and 384 cases in the ORPF arm. The test for heterogeneity indicated *I^2 ^*= 0%, *P *= 0.58. According to a meta-analysis employing a fixed-effects model, there was no difference between both procedures significantly in the occurrence of ANHC [OR = 0.67, 95% CI (0.14, 3.13), *P *= 0.61] (Figure 6).

#### Unaesthetic scar

**Figure 7:**
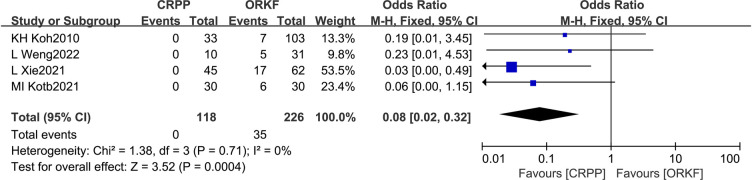
Forest plots of unaesthetic scar.

Four articles (12, 14, 18, 19) reported the occurrence of unaesthetic scars, with 117 cases within the CRPP arm and 226 cases within the ORPF arm; the heterogeneity test revealed *I^2 ^*= 0%, *P *= 0.71; as a result, a fixed-effects meta-analysis was performed, and it revealed that CRPP was superior to ORPP in terms of unaesthetic scars [OR = 0.08, 95% CI (0.02, 0.32), *P *= 0.0004] (Figure 7).

#### LSF

**Figure 8:**
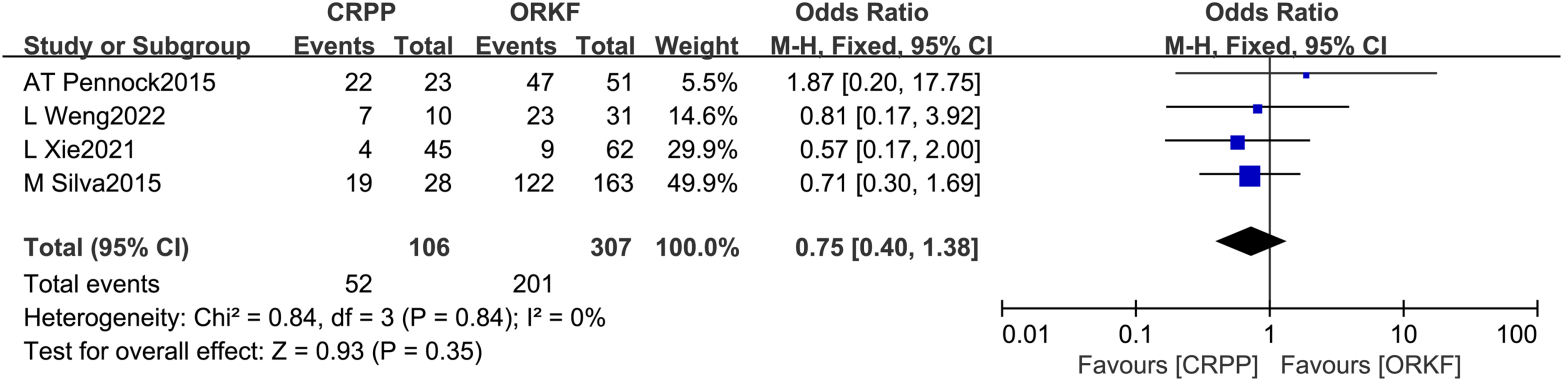
Forest plots of lateral spur formation.

A total of 4 papers (12, 15, 16, 18) reported the occurrence of LSF, with 106 cases within the CRPP arm and 307 cases within the ORPF arm. We found no significant difference in the LSF between both techniques after a fixed-effects meta-analysis based on the heterogeneity test results of *I^2 ^*= 0%, *P *= 0.84 [OR = 0.75, 95% CI (0.40, 1.38), *P *= 0.35] (Figure 8).

## Discussion

HLCF is an intra-articular fracture caused by indirect violence (20), and improper treatment can result in complications such as poor fracture union, cubitus varus or valgus deformity, ANHC, and elbow joint dysfunction due to fracture involvement of the epiphysis (21, 22). CRPP and ORPF are routinely used in patients with severe displacement requiring surgical therapy. However, there is no clinical conclusion as to which of these two treatments is more beneficial, and most research compares them in terms of functional outcomes and complications. Some clinicians argue for using CRPP, claiming that the major drawback of ORPF is the interruption of blood supply following trans-lateral elbow incision, which is deleterious to fracture union and even raises the risk of ANHC (23). However, it has also been suggested that an overemphasis on CRPP and neglect of ORPF might increase soft tissue damage, with higher rates of nerve and vascular injury in some situations of repeated closed repositioning, therefore compromising the postoperative outcome. Consequently, more investigation into the effectiveness and safety of these therapy approaches is required.

There are several fracture classification criteria for pediatric HLCF. Three of the seven included studies used the Song Criteria (12, 14, 18), with all patients having type III-V; three studies used the Jakob Criteria (13, 17, 19), with all patients having type II or higher; and three studies used the Milch Criteria (15, 16, 19), with all fractures having Milch type II. Four of the seven included publications assessed functional outcomes, with two studies employing Hardacre criteria (17, 18), one using Llynn criteria (12), and one without defining evaluation criteria (15). All functional outcomes were rated excellent, good, moderate, or poor. It has been shown that functional recovery of the elbow joint is faster following CRPP, particularly for fractures with displacement less than 4 mm (9, 10, 24), and an excellent and good rate of 98.8% of functional outcomes after CRPP has been reported (25). However, other studies argue that CRPP may impede the functional outcomes for rotated fractures; hence, ORPF is more usually recommended (26), and postoperative joint stiffness and loss of joint mobility may impede the functional outcomes. Nevertheless, functional outcomes and fracture severity were found to be correlated by Changzong Deng et al. (27).

The frequency of excellent and good functional outcomes in the CRPP group was 95.08% for all HLCF children included in this study, which was lower than the literature reported. This study discovered no substantial difference between CRPP and ORPF in functional outcomes, demonstrating that while ORPF may cause more significant soft tissue injury, it does not affect functional outcomes. Both surgical procedures can restore elbow function to an acceptable degree through functional exercise with a sufficiently long follow-up. Functional exercise is believed to improve blood flow in the affected limb, making it more susceptible to trauma repair and fracture union. Stretching and contraction of muscles can activate blood flow in the joint and surrounding tissues, promoting synovial fluid secretion and flow and maintaining normal nutrition of articular cartilage.

Most of the supply of blood for the epiphyseal plate comes from its lateral muscles and soft tissue attachment points such as ligaments, and ORPF will undoubtedly further disrupt fracture blood flow; consequently, it has been learned that ORPF increases the incidence of complications and Iames et al. reported that ORPF could lead to medically induced complications such as poor fracture union, ANHC, and ossifying myositis (28). Kini et al. discovered that the destruction of soft tissues at the fracture site caused complications such as malunion and elbow stiffness, which hindered the functional rehabilitation of the elbow joint during follow-up (29). However, this meta-analysis found no statistical difference between both groups in complications.

There was no statistical difference between the two surgical procedures in the superficial and deep infection prevalence. Despite requiring additional soft tissue damage and incision exposure, ORPF did not enhance the incidence of postoperative infection. Superficial infections, such as pin site and incisional infection, are usually fully recovered with oral antibiotics or Kirschner wire removal. In contrast, managing deep infections, such as septic arthritis and osteomyelitis, is more complicated and can leave a degree of sequelae if not appropriately handled.

Delayed union, nonunion, and malunion may occur following pediatric HLCF, and we refer to these complications as poor fracture union. Poor fracture union can occur for various reasons, including soft tissue damage caused by the fracture or surgery, where blood flow to the fracture block is compromised, and premature postoperative activity leading to fracture instability. Some researchers believe ORPF induces blood supply disruption and raises the risk of poor fracture union (23), hence proposing CRPP. In contrast, several studies suggest that CRPP is more prone to fracture union failure (10). Our study found no statistical difference in the occurrence of poor fracture union between the two interventions. The periosteum and distal fracture block should not be stripped excessively during ORPF. Muscle pulling off the fracture block should be avoided as much as possible (30), as well as the need for a solid external postoperative fixation.

ANHC is a more severe complication of HLCF that can seriously affect functional outcomes. However, its origins are unknown for the time being. ShabtaiLiorl et al. observed an incidence of around 1.4% (31). They identified its key risk factors as high-energy injury and empty repositioning, as well as its morphological and physiological causes as soft tissue stripping and blood supply loss. Skak SV et al. also reported a 0%–7% incidence of ANHC, which can induce consequences such as elbow valgus, which is more challenging to manage (32). Thus, avoidance of ANHC is emphasized. Although CRPP has been shown to decrease the incidence of ANHC (33, 34), In this investigation, we found no statistical difference in the occurrence of ANHC between the two surgical procedures. However, reducing soft tissue injury during surgery may decrease the risk of ANHC.

Due to the requirement of a surgical incision, the ORPF group produced significantly more unaesthetic scars than the CRPP group, which is a benefit of CRPP.

For HLCF, LSF occurs more commonly after surgery. Its incidence has been estimated in the literature to range from 45% to 77%, yet it has little to no effect on functional outcomes. Most of it will be patterned and resorbed later on, and the displacement of the fracture's distal periosteum and the development of new bone may have contributed to its occurrence (35). We do not categorize LSF as a complication since it is virtually universally asymptomatic. In this meta- analysis, we could not identify a substantial difference in LSF incidence rates between the two arms in the current study; thus, we do not believe that the surgical approach influences the development of LSF.

The research does have some flaws, however. (1) The available literature lacks high-quality randomized controlled trials, and the samples of certain studies are small; hence, more randomized controlled studies with larger samples are necessary to provide more credible conclusions. (2) Complications in this Meta-analysis only included infection, poor fracture union, ANHC, and unaesthetic scar. This meta-analysis did not cover more complications, so it was impossible to fully illustrate the benefits and drawbacks of CRPP vs. ORPF in avoiding complications. As a result, the conclusions could have been more reliable. (3) Because CRPP is a novel surgical technique, the varied technical competency of the surgeons may also impact the outcomes and, consequently, compromise the validity of the conclusions of the Meta-analysis.

## Conclusions

For the surgical treatment of pediatric HLCF, this meta-analysis revealed no noticeable difference in effectiveness and safety between CRPP and ORPF. Both surgical procedures were successful, with CRPP having the advantage of the unaesthetic scar.

## Data Availability

The raw data supporting the conclusions of this article will be made available by the authors, without undue reservation.

## References

[1] Wirmer J Kruppa C Fitze G. Operative treatment of lateral humeral condyle fractures in children. Eur J Pediatr Surg. (2012) 22(04):289–94. 10.1055/s-0032-1308709 22570126

[2] Li WC Xu RJ. Comparison of kirschner wires and AO cannulated screw internal fixation for displaced lateral humeral condyle fracture in children. Int Orthop. (2012) 36(6):1261–6. 10.1007/s00264-011-1452-y 22179811 PMC3353087

[3] Landin LA Danielsson LG. Elbow fractures in children. An epidemiological analysis of 589 cases. Acta Orthop Scand. (1986) 57(4):309–12. 10.3109/17453678608994398 3788491

[4] Canavese F Marengo L Tiris A Mansour M Rousset M Samba A Radiological,clinical and functional evaluation using the quick disabilities of the arm,shoulder and hand questionnaire of children with medial epicondyle fractures treated surgically. Int Orthop. (2017) 41(7):1447–52. 10.1007/s00264-017-3442-1 28326443

[5] Zorrilla S Prada-Cañizares A Marti-Ciruelos R Pretell-Mazzini J. Supracondylar humeral fractures in children:current concepts for management and prognosis. Int Orthop. (2015) 39(11):2287–96. 10.1007/s00264-015-2975-4 26311512

[6] Flynn JC Richards JF Saltzman RI. Prevention and treatment of non-union of slightly displaced fractures of the lateral humeral condyle in children. An end-result study. J Bone Joint Surg Am. (1975) 57(8):1087–92. 10.2106/00004623-197557080-00009 1201992

[7] Foster DE Sullivan JA Gross RH. Lateral humeral condylar fractures in children. J Pediatr Orthop. (1985) 5(1):16–22. 10.1097/01241398-198501000-00004 3884662

[8] Fontanetta P Mackenzie DA Rosman M. Missed, maluniting, and malunited fractures of the lateral humeral condyle in children. J Trauma. (1978) 18(5):329–35. 10.1097/00005373-197805000-00006 660686

[9] Song KS Kang CH Min BW Bae KC Cho CH Lee JH. Closed reduction and internal fixation of displaced unstable lateral condylar fractures of the humerus in children. J Bone Joint Surg Am. (2008) 90(12):2673–81. 10.2106/JBJS.G.01227 19047713

[10] Mintzer CM Waters PM Brown DJ Kasserm JR. Percutaneous pinning in the treatment of displaced lateral condyle fractures. J Pediatr Orthop. (1994) 14(4):462–5. 10.1097/01241398-199407000-00008 8077428

[11] Gaston MS Irwin GJ Huntley JS. Lateral condyle fracture of a child's Humerus: the radiographic features may be subtle. Scott Med J. (2012) 57(3):182. 10.1258/smj.2012.012028 22859815

[12] Weng L Cao Y Zhang G Zhou H Liu X Zhang Y. A comparative study on closed reduction vs. Open reduction techniques in the surgical treatment of rotated lateral condyle fractures of the distal humerus in children. Front Pediatr. (2022) 10:891840. 10.3389/fped.2022.891840 35722490 PMC9201398

[13] Justus C Haruno LS Riordan MK Wilsford L Smith T Antekeier S Closed and open reduction of displaced pediatric lateral condyle humeral fractures, a study of short-term complications and postoperative protocols. Iowa Orthop J. (2017) 37:163–9. 28852352 PMC5508286

[14] Kotb MI Alagamy SA Saleh MK. Comparison of closed and open reduction internal fixation of acutely displaced unstable lateral humeral condylar fractures in children: a prospective cohort study. Curr Orthop Pract. (2021) 32(3):261–5. 10.1097/BCO.0000000000000982

[15] Silva M Cooper SD. Closed reduction and percutaneous pinning of displaced pediatric lateral condyle fractures of the humerus: a cohort study. J Pediatr Orthop. (2015) 35(7):661–5. 10.1097/BPO.0000000000000376 25494025

[16] Pennock AT Salgueiro L Upasani VV Bastrom TP Newton PO Yaszay B. Closed reduction and percutaneous pinning versus open reduction and internal fixation for type II lateral condyle humerus fractures in children displaced >2 mm. J Pediatr Orthop. (2016) 36(8):780–6. 10.1097/BPO.0000000000000570 26090985

[17] Liu Y Shi W Zhao H Li Y Li J Xun F Closed reduction and percutaneous pinning versus open reduction and internal fixation for jakob type 3 lateral condyle fractures in children. Int Orthop. (2022) 46(10):2291–7. 10.1007/s00264-022-05476-0 35723700

[18] Xie LW Deng ZQ Zhao RH Wang J Liu X Zhou Y Closed reduction and percutaneous pinning vs open reduction and internal fixation in pediatric lateral condylar humerus fractures displaced by >4mm: an observational cross-sectional study. BMC Musculoskelet Disord. (2021) 22(1):985. 10.1186/s12891-021-04880-8 34823533 PMC8620550

[19] Koh KH Seo SW Kim KM Shim JS. Clinical and radiographic results of lateral condylar fracture of distal humerus in children. J Pediatr Orthop. (2010) 30(5):425–9. 10.1097/BPO.0b013e3181df1578 20574257

[20] Tejwani N Phillips D Goldstein RY. Management of lateral humeral condylar fracture in children. J Am Acad Orthop Surg. (2011) 19(6):350–8. 10.5435/00124635-201106000-00005 21628646

[21] Lan X Dai M Zhang B Huang G. Comparative study of lateral condyle fracture with or without posteromedial elbow dislocation in children. Int Orthop. (2018) 42(3):619–24. 10.1007/s00264-018-3795-0 29392384

[22] Souder CD Roocroft JH Edmonds EW. Significance of the lateral humeral line for evaluating radiocapitellar alignment in children. J Pediatr Orthop. (2017) 37(3):e150–5. 10.1097/BPO.0000000000000853 27603193

[23] Price CT. The treatment of displaced fractures of the lateral humeral condyle in children. J Orthop Trauma. (2010) 24(7):439. 10.1097/BOT.0b013e3181de2d87 20577076

[24] Song KS Shin YW Oh CW Bae KC Cho CH. Closed reduction and internal fixation of completely displaced and rotated lateral condyle fractures of the humerus in children. J Orthop Trauma. (2010) 24(7):434–8. 10.1097/BOT.0b013e3181de014f 20577074

[25] Sahu RL. Percutaneous K wire fixation in pediatric lateral condylar fractures of humerus: a prospective study. Rev Esp Cir Ortop Traumatol (Engl Ed). (2018) 62(1):1–7. 10.1016/j.recot.2017.10.005 29157991

[26] Launay F Leet AI Jacopin S Jouve JL Bollini G Sponseller PD. Lateral humeral condyle fractures in children: a comparison of two approaches to treatment. J Pediatr Orthop. (2004) 24(4):385–91. 10.1097/01241398-200407000-00008 15205620

[27] Deng C Shen Z Wang K Xu W Du W Zhuang W. A novel approach for the treatment of jacob II and III fractures of the lateral humeral condyle in children: percutaneous kirschner wire fixation with ultrasound localization. Front Surg. (2022) 9:1000399. 10.3389/fsurg.2022.1000399 36420410 PMC9676264

[28] Beaty JH Kasser JR. Rockwood and Wilkins’ fractures in children. 6th ed. Philadelphia: Lippincott Williams & Wilkins (2005). 607–10.

[29] Kini SG Arora A Kumar S Mehra A. Lateral condylar fractures of the humerus in children-A 13 year follow up study. SICOT. (2009).

[30] Wattenbarger JM Gerardi J Johnston CE. Late open reduction internal fixation of lateral condyle fractures. J Pediatr Orthop. (2002) 22(3):394–8. 10.1097/01241398-200205000-00026 11961463

[31] Shabtai L Lightdale-Miric N Rounds A Arkader A Pace JL. Incidence, risk factors and outcomes of avascular necrosis occurring after humeral lateral condyle fractures. J Pediatr Orthop B. (2020) 29(2):145–8. 10.1097/BPB.0000000000000698 31821269

[32] Skak SV Olsen SD Smaabrekke A. Deformity after fracture of the lateral humeral condyle in children. J Pediatr Orthop B. (2001) 10(2):142–52. 10.1097/01202412-200104000-00012 11360781

[33] Gendi K Livermore A Browne J Machurick M Halanski MA Noonan KJ. Open vs. Closed reduction in type 2 lateral condyle fractures. Iowa Orthop J. (2019) 39(1):51–5. 31413674 PMC6604542

[34] Shaerf DA Vanhegan IS Dattani R. Diagnosis,management and complications of distal humerus lateral condyle fractures in children. Shoulder Elbow. (2018) 10(2):114–20. 10.1177/1758573217701107 29560037 PMC5851120

[35] Pribaz JR Bernthal NM Wong TC Silva M. Lateral spurring (overgrowth) after pediatric lateral condyle fractures. J Pediatr Orthop. (2012) 32(5):456–60. 10.1097/BPO.0b013e318259ff63 22706459

